# Gut microbiota, inflammatory proteins and COVID-19: a Mendelian randomisation study

**DOI:** 10.3389/fimmu.2024.1406291

**Published:** 2024-05-13

**Authors:** Yuling Chen, Chang Chen

**Affiliations:** ^1^ Department of Clinical Laboratory, Nanchong Central Hospital (Nanchong Hospital of Beijing Anzhen Hospital, Capital Medical University), The Second Clinical Medical College of North Sichuan Medical College, Nanchong, Sichuan, China; ^2^ Medical Department, Nanchong Guoning Mental Health Hospital, Nanchong, Sichuan, China

**Keywords:** COVID-19, gut microbiota, inflammatory proteins, Mendelian randomization, meta

## Abstract

**Background:**

The human gut microbiota has been identified as a potentially important factor influencing the development of COVID-19. It is believed that the disease primarily affects the organism through inflammatory pathways. With the aim of improving early diagnosis and targeted therapy, it is crucial to identify the specific gut microbiota associated with COVID-19 and to gain a deeper understanding of the underlying processes. The present study sought to investigate the potential causal relationship between the gut microbiota and COVID-19, and to determine the extent to which inflammatory proteins act as mediators in this relationship.

**Methods:**

Bidirectional mendelian randomization (MR) and Two-step mediated MR analyses were applied to examine causative associations among 196 gut microbiota, 91 inflammatory proteins and COVID-19. The main analytical method used in the MR was the random effects inverse variance weighted (IVW) method. This was complemented by the Bayesian weighted Mendelian randomization (BWMR) method, which was utilized to test the hypothesis of MR. In order for the results to be deemed reliable, statistical significance was required for both methods. Validation was then carried out using an external dataset, and further meta-analyses were conducted to authenticate that the association was reliable.

**Results:**

Results of our research indicated that seven gut microbiota were actively associated to the COVID-19 risk. Five inflammatory proteins were associated with COVID-19 risk, of which three were positively and two were negatively identified with COVID-19. Further validation was carried out using sensitivity analyses. Mediated MR results revealed that CCL2 was a possible mediator of causality of family Bifidobacteriaceae and order Bifidobacteriales with COVID-19, mediating at a ratio of 12.73%.

**Conclusion:**

Suggesting a genetic causation between specific gut microbiota and COVID-19, our present research emphasizes the underlying mediating role of CCL2, an inflammatory factor, and contributes to a deeper understanding of the mechanism of action underlying COVID-19.

## 1 Introduction

Coronavirus disease 2019 (COVID-19) is a hugely transmissible viral illness of the respiratory tract due to the SARS-CoV-2 virus, which deals a far-reaching blow to global health systems, society, and economies of development (1). SARS-CoV-2 is part of the genus β-coronavirus (2) that infects host cells through its spiking protein combined with angiotensin-converting enzyme 2 (ACE 2) (3, 4). Immediately entering into the host cell, genomic RNA of SARS-CoV-2 is mobilized and begins to replicate. New viroids then assemble and are secreted into the cell interstitium to infect neighboring cells (5).

Symptoms most frequently reported with COVID-19 are dry cough, fever, and muscle pain, with accumulation of other systems such as cardiovascular, neurological, dermatological, and gastrointestinal systems also occurring (6, 7). COVID-19 patients reported a wide range of gross intestinal symptoms including disgust, emesis, diarrhea, lack of appetite and abdominal pain (8). Also, in addition to the lung, ACE 2 has been described as highly expressed in the gastrointestinal tract (9, 10). Even, in some case reports, it has been found that gastrointestinal symptoms may have occurred before respiratory symptoms (11). Respiratory viral infections proved to be correlated to changes of the components within the intestinal microbiota (12), and in COVID-19, modifications in the gut microbiota may play a role by undermining host immune homeostasis (13). It is therefore possible to postulate that in COVID-19, crosstalk may occur between gut microbiota and lung inflammation, which may contribute to the pathogenesis of the disease.

The hyperinflammatory reaction triggered when SARS-CoV-2 gains access to pulmonary alveolar epithelium via ACE 2 (also known as cytokine storm) has been a key driver in the pathogenesis of COVID-19 (14). Findings suggest that many inflammatory cytokines are altered in COVID-19 and that its elevated levels correlate with disease severity (15, 16). Hence, a thorough study into the association among the gut microbiota, inflammatory proteins and COVID-19 may expand what we know about the pathogenesis of the disease, as well as potentially offer new biomarkers and curative targets based on the microbiome or inflammatory proteins for COVID-19.

Mendelian randomization (MR) is an approach for assessing causal effects by exposure on outcomes utilizing single nucleotide polymorphisms (SNPs) as a genetic tool variable (17). Since genetic variants are distributed randomly, it follows that MR studies are less vulnerable to the effects of confounding factors than traditional observational studies (18). Bayesian weighted Mendelian randomization (BWMR) represents a statistical inference method that addresses the issue of uncertainty in the presence of weak effects resulting from polygenicity and the violation of the IV hypothesis as a consequence of multidirectionality. The method employs Bayesian weighted detection of outliers in order to provide a robust and reliable approach to identifying such effects (19). We aimed to establish whether gut microbiota and COVID-19 are causally related and to assess the degree to which inflammatory proteins mediate the influence exerted by the gut microbiota upon COVID-19. The main methodology employed in this study is that of MR analysis, which is divided into two main categories: bidirectional MR and two-step MR analysis. Bidirectional MR analysis is a positive and negative MR analysis with gut microbiota and COVID-19 set as exposure and outcome, respectively. In order to satisfy the mediation analysis, it is necessary to ensure that the factors cannot be both cause and effect. That is to say, gut microbiota has an effect on COVID-19, and in turn, COVID-19 has no effect on gut microbiota. Two-step MR analyses are employed to evaluate potential intermediate variables of causal associations, with the objective of exploring whether the gut microbiota contributes to disease development through intermediate variables. In the initial step, the association between the gut microbiota and intermediate variables is calculated by MR. In the subsequent stage, the association between intermediate variables and COVID-19 is calculated by MR. Finally, the direct and indirect effects between the gut microbiota and COVID-19 are integrated and calculated (20).

## 2 Materials and methods

### 2.1 Study design

**Figure 1:**
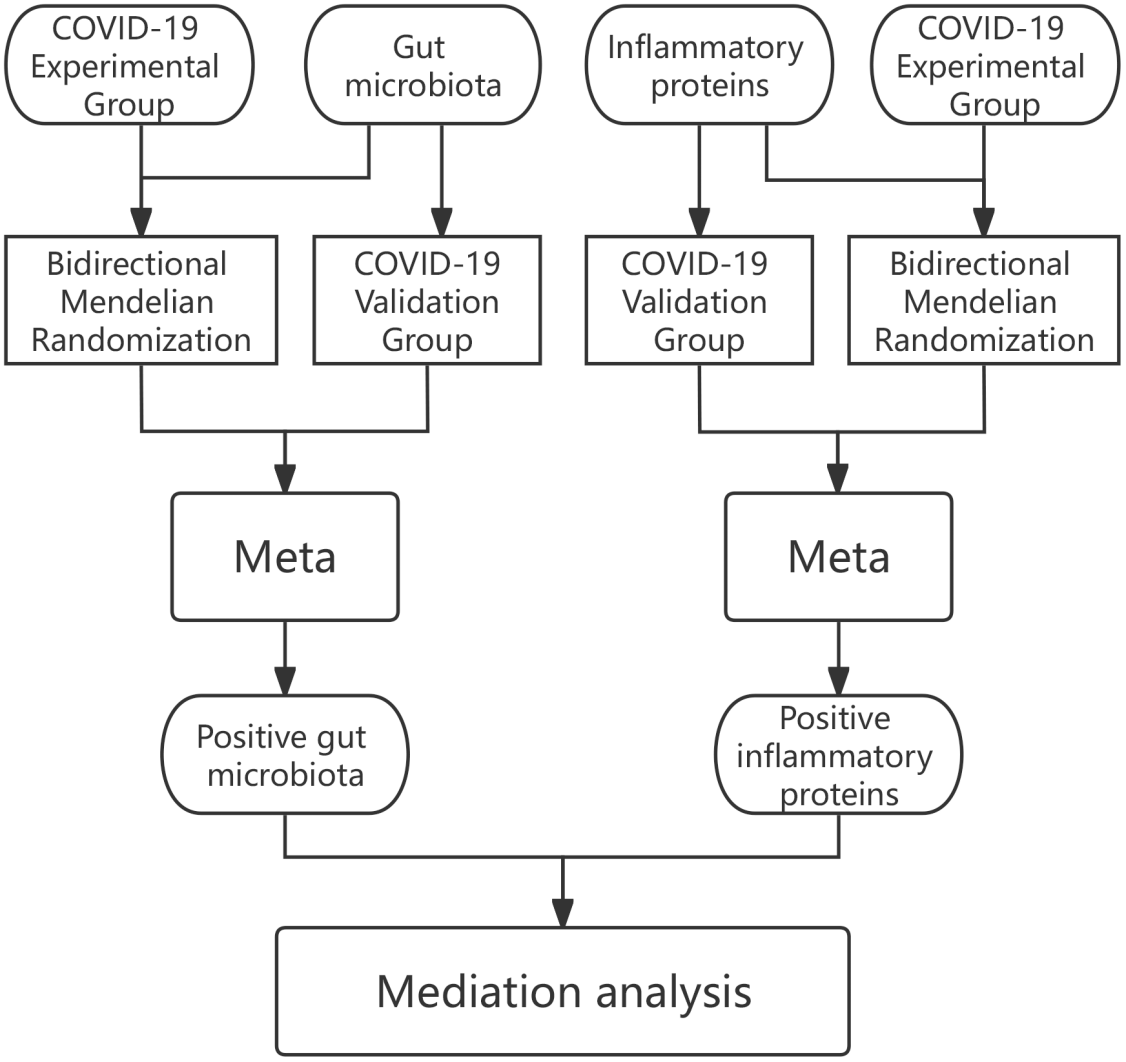
Experimental procedure for this study.

In our analyses we adopted data were obtained from openly accessible shared repositories and so there has been nothing to request in terms of extra ethics clearance for the present study. the cause-and-effect relationship that exists within the gut microbiota in relation to COVID-19 was first explored in the first part of the research by bidirectional MR, and positive gut microbiota were screened by Meta-analysis of experimental and validation group results. The positive proteins were then screened among 91 inflammatory proteins using the same method. After this, the causal relationship between specific gut microbiota mediated by inflammatory proteins and COVID-19 was investigated in depth using mediated MR (Two-step MR) (**Figure 1**).

### 2.2 Data sources

All figures utilized as part of our survey were publicly accessible and the participants in the study were all individuals of European ancestry. Gut microbiota figures taken directly form the MiBioGen Consortium (The data could be downloaded from the homepage of the official website at the following URL: https://mibiogen.gcc.rug.nl/). Inflammatory proteins data were extracted from the EBI GWAS Catalog, accession numbers GCST90274758 to GCST90274848 (The data could be downloaded from the homepage of the official website by entering the corresponding number at the following URL: https://www.ebi.ac.uk/gwas/home) (21). COVID-19 experimental group data were taken from COVID-19 Host Genetics (https://www.covid19hg.org/results/r7/) comprising 122,616 patients and 247,540 controls. Data for the COVID-19 validation set were derived out of the FinnGen Alliance data source (https://www.finngen.fi/en) (22) and consisted of 2856 cases and 405,232 controls.

### 2.3 Genetic selection

After removing the unnamed bacteria, we got a total of 196 gut microbiota. TwoSampleMR was used to screen the SNPs (23). The significance threshold was relaxed and set to p<1e-5 in order to satisfy that all gut microbiota and inflammatory proteins had a moderate number of SNPs (24). Clustering of those SNPs were then performed according to the interlocking imbalances (kb= 10,000 kb and r2 = 0.001). Determining the existence of weak instrumental variable bias, an F-statistic was computed in order to measure the power level for the indicator variant, with an F-statistic above 10 meaning that there was a low likelihood that the indicator variant would be weakly biased (25).

### 2.4 MR analysis

MR analyses were combined with the “mr” functionality as well as with five methodologies: MR Egger, Weighted Median, Inverse Variance Weighted (IVW), Simple Mode and Weighted Mode. Central focus of this study was the results of the IVW method, which showed the strongest ability to test for causal relationships in MR analyses (26). In the event that the IVW method yielded a p-value of less than 0.05, the result was incorporated into the subsequent study (27). Subsequently, BWMR was employed to reinforce the IVW method, with the outcome deemed significant when both methods yielded p-values below 0.05. The integration of these two methodologies could facilitate a more precise assessment of the influence of risk factors on complex traits or diseases. For each MR, a calibration for false discovery rate (FDR) was implemented and the FDR threshold was set at q<0.1. When p<0.05 and q≥0.1, the result was regarded as suggestive of a correlation (28). Then the odds ratio (OR) was calculated, where more than 1 was a danger element and less than 1 was a protection element. To assure the precision of the analyses, several kinds of susceptibility tests, consisting of heterogeneity tests, horizontal multivariate tests, as well as leave-one-out sensitivity tests (LOO), were employed in order to fully assess the reliability concerning these findings.

### 2.5 Intermediary effect

We employed a Two-step MR approach to intermediation analysis, through which the total effect was able to be disaggregated into a direct effect and an indirect effect (20). The total impact with respect to the gut microbiota upon COVID-19 was disaggregated into its direct effect of gut microbiota on COVID-19, and an indirect effect mediated through mediators. Calculating what proportion of the effect was mediated was done through division of the indirect benefit by the total benefit.

## 3 Results

### 3.1 MR results of gut microbiota and COVID-19

**Figure 2:**
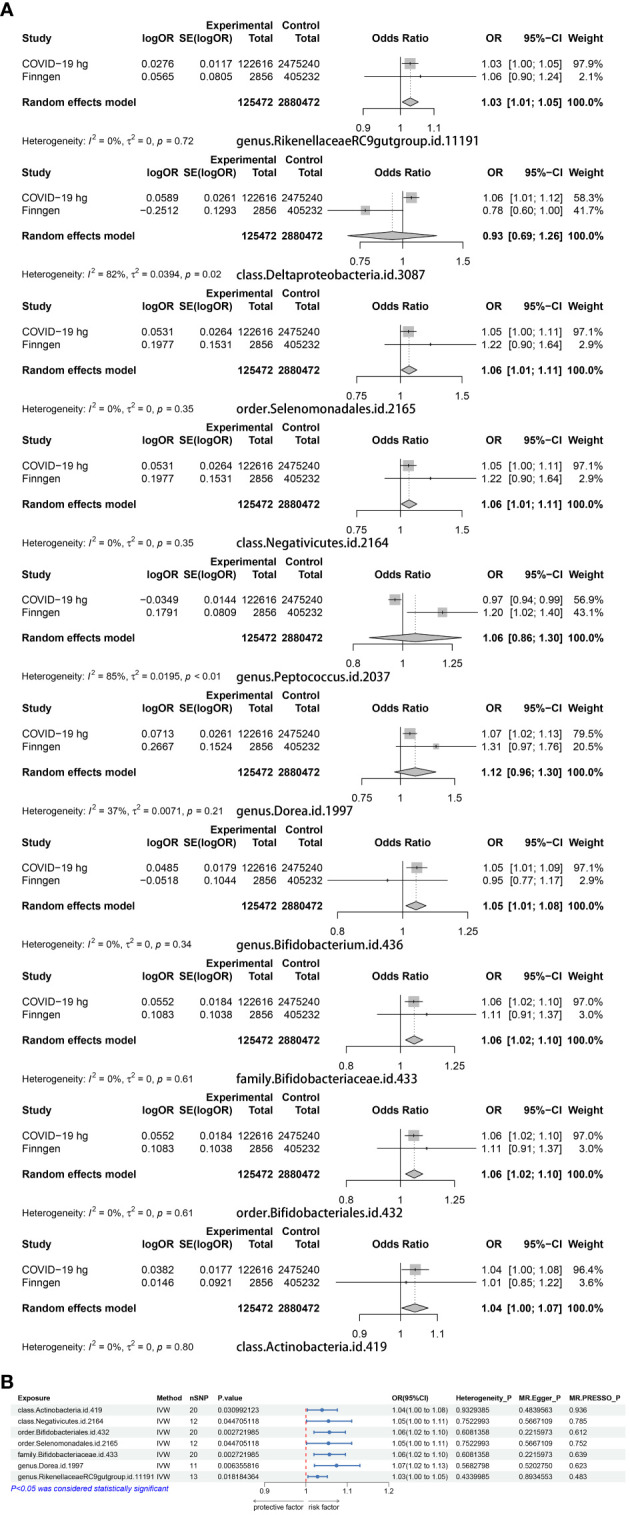
**(A)** A Meta-analysis of the results of the experimental group of 10 gut microbiota and the results of the validation group. **(B)** MR results of the causal relationship between seven positive gut microbiota and COVID-19.

We examined MR correlations relating 196 gut microbiota to the COVID-19 experimental group, with a total of 12 suggesting a correlation (**Supplementary Table 1**). After BWMR tests were performed, 2 groups of gut microbiota were screened out (**Supplementary Table 2**). The 10 gut microbiota were subjected to MR analysis with the COVID-19 validation group and the results were meta-merged with the experimental group (**Figure 2A**; **Supplementary Table 3**). Finally, seven positive gut microbiota were obtained that were positively correlated with COVID-19, which were class Actinobacteria (OR = 1.05, 95% CI: 1.00–1.10, p = 0.036), class Negativicutes (OR = 1.05, 95% CI: 1.00–1.11, p = 0.045), order Bifidobacteriales (OR = 1.06, 95% CI: 1.02–1.10, p = 0.003), order Selenomonadales (OR = 1.05, 95% CI: 1.00–1.11, p = 0.045), family Bifidobacteriaceae (OR = 1.06, 95% CI: 1.02–1.10, p = 0.003), genus Dorea (OR = 1.07, 95% CI: 1.02–1.13, p = 0.006), genus RikenellaceaeRC9gutgroup (OR = 1.03, 95% CI: 1.00–1.05, p = 0.018), suggesting they may be risk factors for COVID-19. To verify the above results, we carried out sensitivity analyses (**Figure 2B**; **Supplementary Figure S1**).

### 3.2 MR results of inflammatory proteins and COVID-19

**Figure 3:**
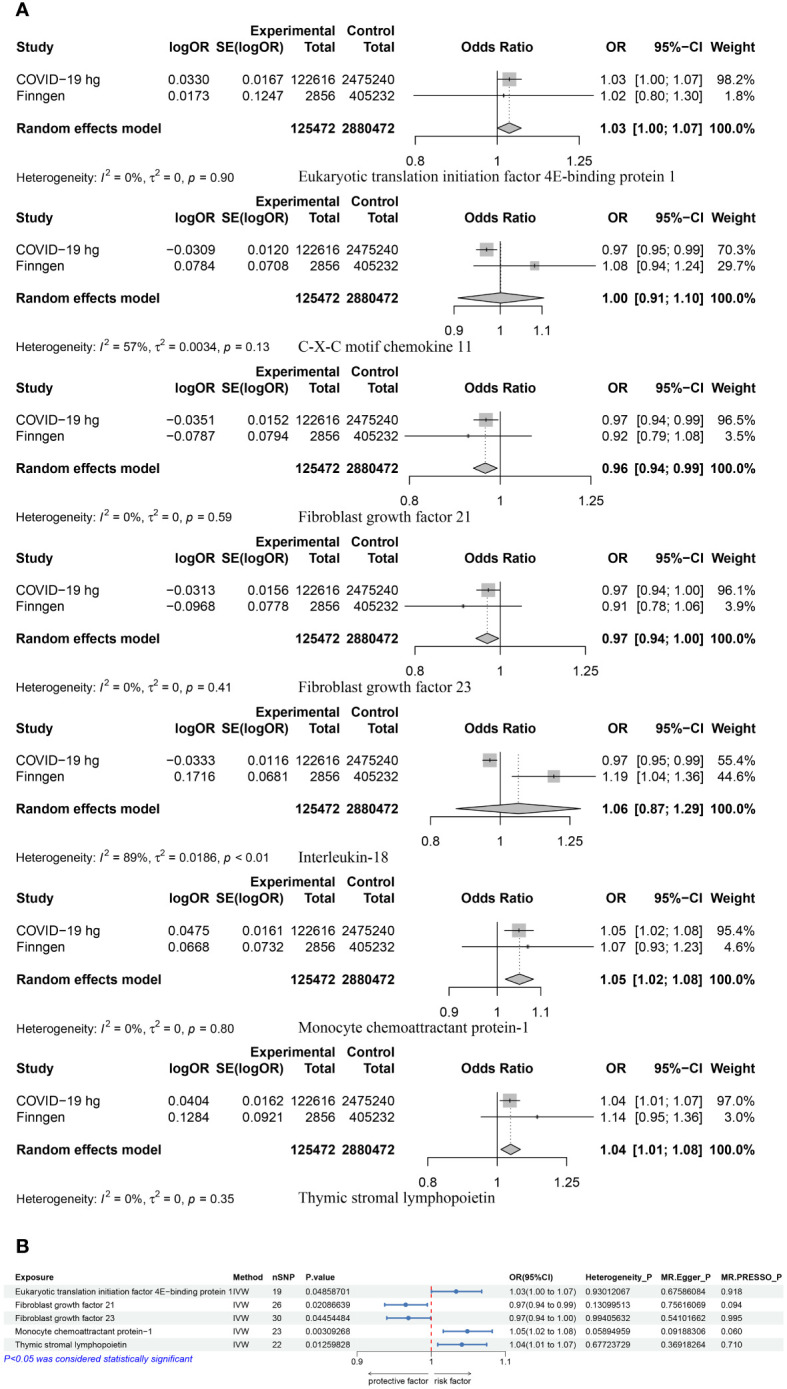
**(A)** A Meta-analysis of the results of the experimental and validation groups for seven inflammatory proteins. **(B)** MR results for the causal association of five positive proteins with COVID-19.

MR results of 91 inflammatory proteins and the COVID-19 experimental group suggested that there was a correlation between a total of 10 proteins (**Supplementary Table 4**), of which Leukemia inhibitory factor receptor (LIF-R) passed the FDR correction (P=0.034). Three proteins were screened out after performing BWMR tests (**Supplementary Table 5**). The seven inflammatory proteins were subjected to Mendelian randomization analysis with the COVID-19 validation group and the results were Meta-merged with the experimental group (**Figure 3A**; **Supplementary Table 6**), resulting in five positive inflammatory proteins associated with COVID-19, which included Eukaryotic translation initiation factor 4E- binding protein 1 (4EBP1) (OR = 1.03, 95% CI: 1.00–1.07, p = 0.049), Monocyte chemoattractant protein-1 (MCP-1/CCL2) (OR = 1.05, 95% CI: 1.02–1.08, p = 0.003), Thymic stromal lymphopoietin (TSLP) (OR = 1.04, 95% CI: 1.01–1.07, p = 0.013) was positively correlated with COVID-19, and Fibroblast growth factor 21 (FGF-21) (OR = 0.97, 95% CI: 0.94–0.99, p = 0.021), Fibroblast growth factor 23 (FGF-23) (OR = 0.97, 95% CI: 0.94–1.00, p = 0.045) were negatively correlated with COVID-19. Sensitivity analyses were performed in order to verify of the above findings (**Figure 3B**; **Supplementary Figure S2**).

### 3.3 MR results of gut microbiota and inflammatory proteins

**Figure 4:**
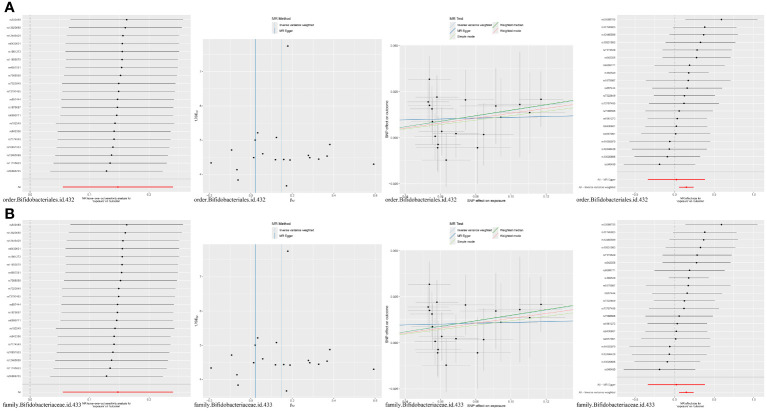
**(A)** MR results for the causal association of order Bifidobacteriales with CCL2. **(B)** MR results for the causal association of family Bifidobacteriaceae with CCL2.

MR analysis of seven gut microbiota and five inflammatory proteins was performed, which suggested that family Bifidobacteriaceae (OR = 1.16, 95% CI: 1.06–1.27, p = 0.0018) and order Bifidobacteriales (OR = 1.16, 95% CI: 1.06–1.27, p = 0.0018) may be risk factors for CCL2 (**Table 1**, **Figure 4**). Sensitivity analyses were also conducted to confirm this result (**Table 1**, **Figure 4**).

**Table 1:** MR results of gut microbiota and inflammatory proteins.

Exposure	Outcome	Method	nSnp	pval	Beta	Heterogeneity P	MR-Egger P	MR-PRESSO P
Family Bifidobacteriaceae	CCL2	MR Egger	20	0.903	0.023	0.786		
		Weighted median	20	0.023	0.150			
		IVW	20	0.002	0.148	0.808	0.493	0.665
		Simple mode	20	0.308	0.120			
		Weighted mode	20	0.216	0.133			
Order Bifidobacteriales	CCL2	MR Egger	20	0.903	0.023	0.730		
		Weighted median	20	0.026	0.150			
		IVW	20	0.002	0.148	0.738	0.493	0.652
		Simple mode	20	0.341	0.120			
		Weighted mode	20	0.207	0.133			

### 3.4 Intermediary effect

Two-step MR and bidirectional MR analyses were performed to examine the mediating pathways from order Bifidobacteriales through CCL2 to COVID-19, and family Bifidobacteriaceae through CCL2 to COVID-19. The bidirectional MR results demonstrated that there was no causal effect of COVID-19 on the order Bifidobacteriales and family Bifidobacteriaceae (**Table 2**). The Two-step MR results indicated that CCL2 may act as a mediator in the causal relationship between family Bifidobacteriaceae and order Bifidobacteriales and COVID-19, with a mediation ratio of 12.73% (**Table 3**).

**Table 2:** COVID-19 with order Bifidobacteriales and family Bifidobacteriaceae inverse MR results.

Outcome	Exposure	Method	nsnp	pval
COVID-19	order Bifidobacteriales	MR Egger	14	0.7455804
		Weighted median	14	0.791070751
		Inverse variance weighted	14	0.623264111
		Simple mode	14	0.989716363
		Weighted mode	14	0.966752385
COVID-19	family Bifidobacteriaceae	MR Egger	14	0.7455804
		Weighted median	14	0.791945159
		Inverse variance weighted	14	0.623264111
		Simple mode	14	0.989813047
		Weighted mode	14	0.963279845

**Table 3:** Mediation of COVID-19 by gut microbiota through inflammatory proteins.

Exposure	Mediator	Total effect	Mediation effect	Direct effect	Mediated proportion(%) (95%CL)
order Bifidobacteriales	CCL2	0.055	0.007	0.048	12.73 (-12.23–37.68)
family Bifidobacteriaceae	CCL2	0.055	0.007	0.048	12.73 (-12.23–37.68)

## 4 Discussion

In this comprehensive mediated MR study, we identified seven gut microbiota (class Actinobacteria, class Negativicutes, order Bifidobacteriales, order Selenomonadales, family Bifidobacteriaceae, genus Dorea, genus RikenellaceaeRC9gutgroup) and five inflammatory proteins (4EBP1, CCL2, TSLP, FGF-21, FGF-23) in a causal relationship to COVID-19. Mediated MR results indicated that CCL2 may be a mediator of the causal relationship between family Bifidobacteriaceae and order Bifidobacteriales to COVID-19, with a mediation ratio of 12.73%. This analysis highlighted the link from the gut microbiota to COVID-19, highlighting the mediating role of the inflammatory protein CCL2.

The gastrointestinal system is considered the largest immunological apparatus in the human body, and its residing gut microbiota regulate host immune system, ward off pollutants and provide nutritional metabolism support (29). Gut microbiota play a crucial part in health and illnesses in humans. Researchers have found that gut microbiota affect lung health through an important crosstalk with the lungs, the “gut-lung axis” (30). Gut ecological dysregulation can lead to increased vulnerability to respiratory diseases (31). Gut-Lung axis is bi-directional; gut microbiota metabolites can affect the lungs through the bloodstream and it can also affect the gut microbiota when inflammation occurs in the lungs (32).

The human gut microbiota is a complex system of microorganisms, including bacteria, fungi, archaea, and viruses. It has been shown that the dynamics of the gut microbiota are associated with the severity of COVID-19. However, there is a lack of understanding of the role that fungi and viruses play in the gut microbiota in COVID-19 infections, as the majority of previous studies have focused on the more abundant bacterial component (33). A limited number of studies have indicated a correlation between SARS-CoV-2 infection and changes to the composition of fungal microbiomes (34). In severe cases of COVID-19, an increase in Candida albicans has been observed, and gastrointestinal symptoms, such as persistent diarrhea, in COVID-19 patients may result from an inflammatory response triggered by the virus (35). Nevertheless, the current study yielded disparate outcomes at the bacterial level due to a limited sample size and inconsistent patient severity. Some studies have identified phylum Actinobacillus as the dominant phylum in patients with COVID-19 and healthy controls. whereas genus Dorea was found to be significantly reduced (36). Furthermore, studies on genus Dorea have indicated that genus Dorea is highly associated with mortality in patients with COVID-19 (37). Some scholars have found that patients with COVID-19 have a significantly reduced number of bifidobacteria (38, 39). It was then postulated that beneficial probiotics, such as bifidobacteria, are employed to orchestrate changes in the gut microbiota in order to prevent or treat disease progression (40, 41). A randomized controlled trial conducted in Hong Kong demonstrated that oral administration of Bifidobacterium reduced the incidence of SARS-CoV-2 infection and improved the clinical outcome (42). Nevertheless, it has been reported that there was no significant difference in the expression of bifidobacterial abundance in patients with COVID-19, with the exception of those in the ICU (43). Furthermore, no differences in probiotic abundance were found at any stage of the viral infection (44). A study conducted on a European population also demonstrated an increased expression of bifidobacterial abundance in individuals infected with SARS-CoV-2 (45). This suggests that racial differences between individuals may greatly influence the gut microbiome. The majority of current studies have focused on adult populations, with relatively few studies investigating the impact of SARS-CoV-2 on the gut microbiome in children. Nevertheless, a reduction in the abundance of bifidobacteria has also been reported in pediatric patients (46). A more consistent conclusion is that the relative abundance of Actinobacteria is significantly higher in patients with COVID-19 (47, 48).

The present study focused on microorganisms at the genus level or above. However, species and subspecies within any genus may have different or opposing effects on host metabolism. For instance, Bifidobacterium longum was significantly enriched in disease, and Bifidobacterium animals was positively correlated with the severity of COVID-19 (36). However, Bifidobacterium longum was also found to be significantly enriched in the healthy group compared to patients with COVID-19 (41). Furthermore, Bifidobacterium longum was reduced in non-diarrheal patients with complications associated with COVID-19 (39). The results of this study were predicated upon a theoretical framework of genetic inheritance, with further validation in larger cohorts required to substantiate this hypothesis. Conversely, although the prevailing view is that prebiotics, including bifidobacteria, are generally safe, there are dozens of cases in which they have been found to cause bifidobacterial bacteremia (49). The majority of these patients exhibited either gastrointestinal-related symptoms or were immunocompromised (50, 51). It is documented that Bifidobacterium longum, Bifidobacterium shortum, and Bifidobacterium animalis are the primary Bifidobacteria that cause bacteremia in European populations. The speculation is that the cause may be attributed to gastrointestinal disorders resulting in mucosal damage and intestinal leakage, allowing the bacteria to translocate from the gut to the bloodstream, thereby triggering an infection (52). This indicates that, although Bifidobacterium bifidum is typically regarded as a safe bacterium, it is crucial to maintain a high degree of awareness concerning its potential toxicity, antimicrobial resistance, and adverse metabolic activity, particularly in patients with impaired immune systems (51, 52).

One of the mechanisms of COVID-19 pathogenesis is to cause a cytokine storm. It is a fatal generalized inflammatory response which involves mainly autocrine and paracrine activation of leukocytes, macrophages, endothelial cells, and mast cells, associated with the release for high levels of pro-inflammatory cytokines and chemokines in COVID-19, including IL-2, IL-6, IL-8, IL-10, IL-18, IP-10, CCL 2, IFN-γ, and TNF-α (53, 54). CCL2 is an essential mediator of inflammation that is essential for the management of viral infections (55). Dysregulated gut ecology has been found to be associated with increased mortality from respiratory infections, possibly due to dysregulated immune responses, increased CCL2 secretion, and reduced numbers of lung regulatory T cells (56). Evidence suggests that an involvement of CCL2 and its receptor (CCR2) is involved in the collection of monocytes and the infusion of these cells into the lungs of COVID-19 patients. At the same time, the massive secretion of CCL2 after infection leads to an excessive inflammatory response in the organism (57). In this regard, there is a possibility that CCL 2 may be one of the major causes of serious infections and even death in COVID-19 (58). Our study found that CCL 2 was one of the risk elements not only for COVID-19, but also played a mediating role in the pathogenic impact of intestinal microbiota on COVID-19. Nevertheless, the precise mechanism by which family Bifidobacteriaceae and order Bifidobacteriales contribute to the pathogenesis of COVID-19 via CCL2 remains unclear and requires further investigation. This represents a potential breakthrough in future research.

Here are the observations from the first large-scale MR analyses of the cause-and-effect associations among gut microbiota, inflammatory proteins, and COVID-19. Our preliminary findings suggested a cause-and-effect connection with the gut microbiota in relation to COVID-19, in which inflammatory proteins may play a mediating role, which provided novel thoughts for the therapy and prevention for COVID-19. However, our study had a number of limitations. Firstly, participation in this were European populations, which may produce racial differences. Second, the results were not corrected for FDR and they were suggestive, which requires further validation with cellular, animal and clinical experiments.

## 5 Conclusion

Our MR studies suggested potential causal relationships between gut microbiota, inflammatory proteins, and COVID-19. Specifically, CCL2 mediated the regulatory effects of family Bifidobacteriaceae and order Bifidobacteriales on COVID-19. Our findings provided genetic evidence for the immune mechanism of COVID-19 and offered new possibilities in the area of disease avoidance and therapy.

## Data availability statement

The original contributions presented in the study are included in the article/**Supplementary Material**. Further inquiries can be directed to the corresponding author.

## Ethics statement

Ethical approval was not required for the study involving humans in accordance with the local legislation and institutional requirements. Written informed consent to participate in this study was not required from the participants or the participants’ legal guardians/next of kin in accordance with the national legislation and the institutional requirements.

## Author contributions

YC: Conceptualization, Funding acquisition, Investigation, Methodology, Project administration, Resources, Validation, Visualization, Writing – original draft, Writing – review & editing. CC: Conceptualization, Data curation, Formal Analysis, Software, Validation, Writing – review & editing.
