# Supplementary material for: Gut microbiota, inflammatory proteins and COVID-19: a Mendelian randomisation study
Source: Front Immunol. 2024 May 13;15:1406291. doi: 10.3389/fimmu.2024.1406291 (PMC11128586; doi:10.3389/fimmu.2024.1406291)
Supplement: Supplementary file 2 [file Image_1.pdf]

S1. Resultant plots of 7 suggestive gut microbiota associated with COVID-19. (A), class.Actinobacteria (B), class.Negativicutes (C), family.Bifidobacteriaceae (D), genus.Dorea (E), genus.RikenellaceaeRC9gutgroup (F), order.Bifidobacteriales (G), order.Selenomonadales

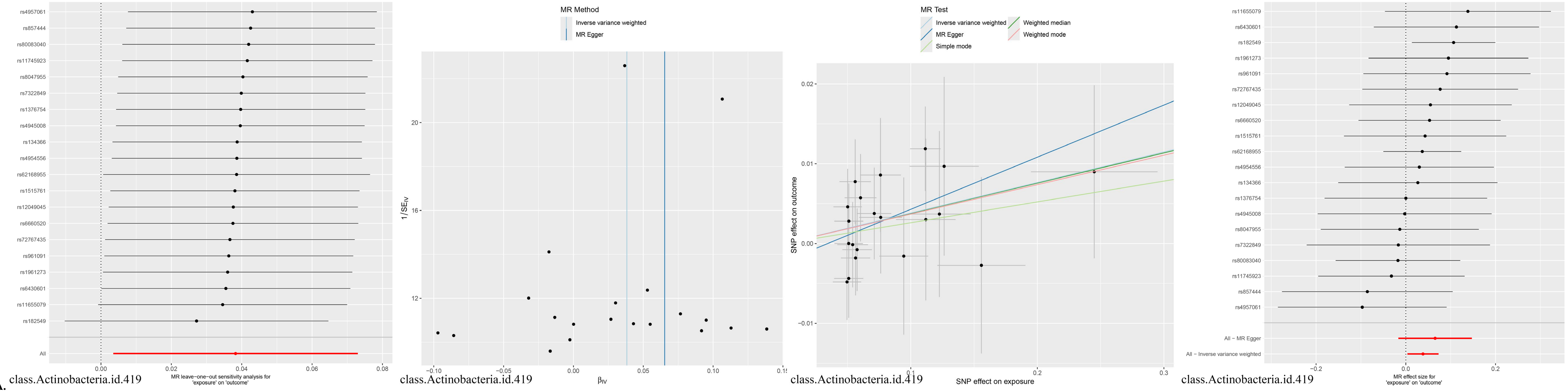

A.

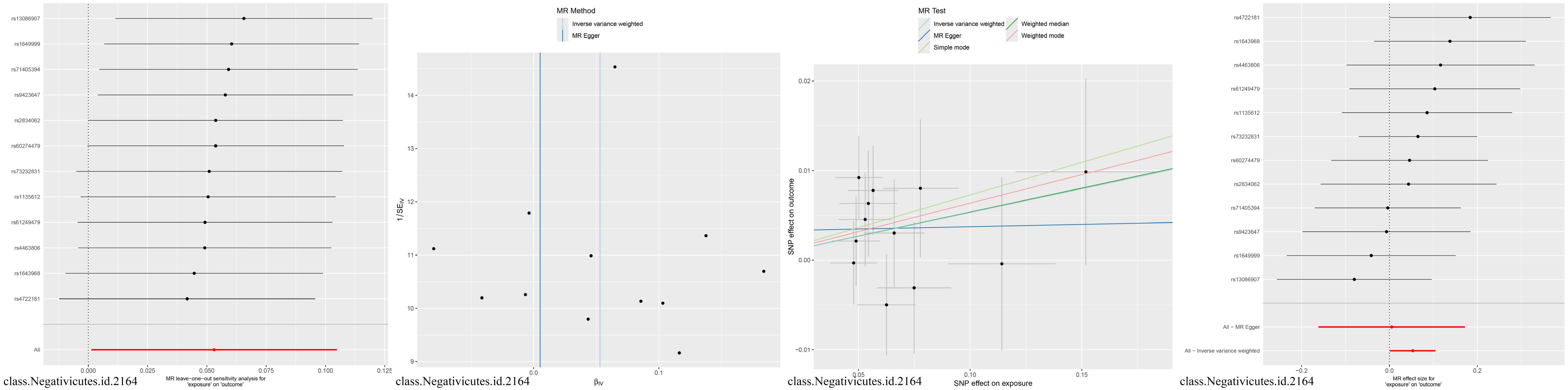

MR leave−one−out sensitivity analysis for 'exposure' on 'outcome'
class.Negativicutes.id.2164
MR Method
Inverse variance weighted
MR Egger
1/SE_IV
β_IV
class.Negativicutes.id.2164
MR Test
Inverse variance weighted
MR Egger
Simple mode
Weighted median
Weighted mode
SNP effect on outcome
SNP effect on exposure
class.Negativicutes.id.2164
All − MR Egger
All − Inverse variance weighted
MR effect size for 'exposure' on 'outcome'
class.Negativicutes.id.2164

B.

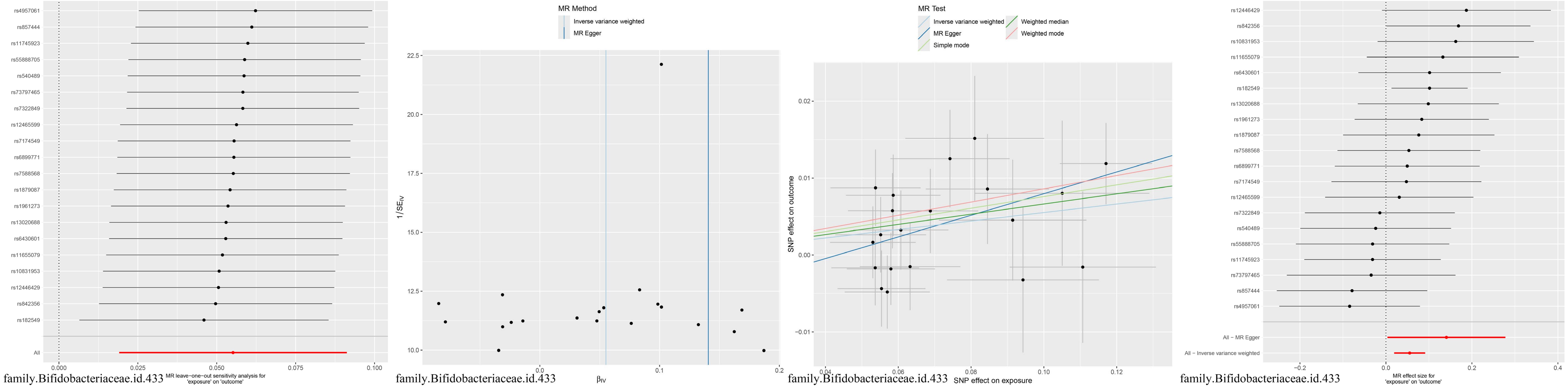

MR leave−one−out sensitivity analysis for 'exposure' on 'outcome'
family.Bifidobacteriaceae.id.433
MR Method
Inverse variance weighted
MR Egger
1/SE_IV
β_IV
family.Bifidobacteriaceae.id.433
MR Test
Inverse variance weighted
MR Egger
Simple mode
Weighted median
Weighted mode
SNP effect on outcome
SNP effect on exposure
family.Bifidobacteriaceae.id.433
All − MR Egger
All − Inverse variance weighted
MR effect size for 'exposure' on 'outcome'
family.Bifidobacteriaceae.id.433

C.

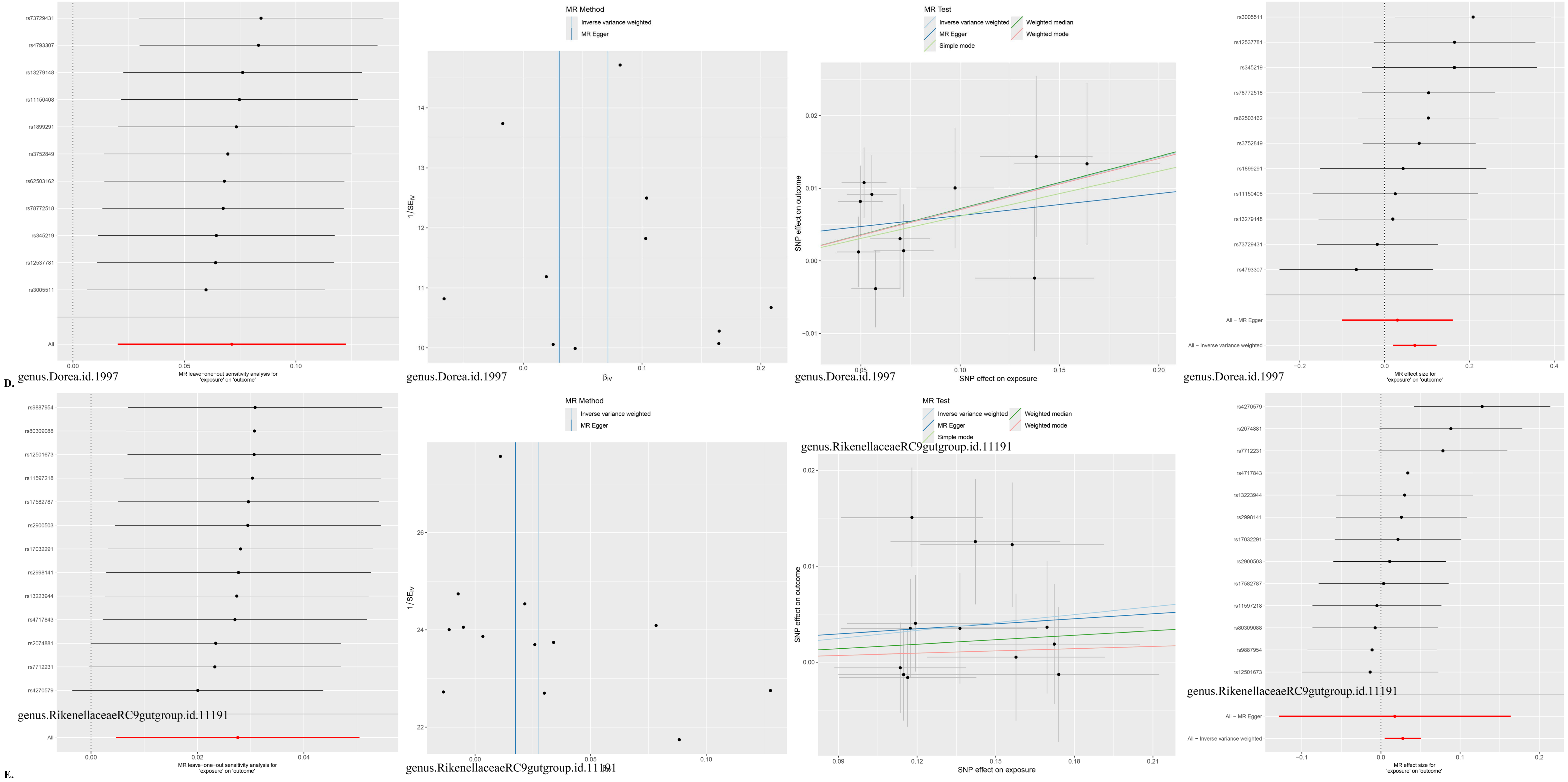
D.
genus.Dorea.id.1997
MR leave−one−out sensitivity analysis for 'exposure' on 'outcome'
rs73729431
rs4793307
rs13279148
rs11150408
rs1899291
rs3752849
rs62503162
rs78772518
rs345219
rs12537781
rs3005511
All
MR Method
Inverse variance weighted
MR Egger
1/SE_IV
β_IV
MR Test
Simple mode
Weighted median
Weighted mode
SNP effect on outcome
SNP effect on exposure
All − MR Egger
All − Inverse variance weighted
MR effect size for 'exposure' on 'outcome'
E.
genus.RikenellaceaeRC9gutgroup.id.11191
rs9887954
rs80309088
rs12501673
rs11597218
rs17582787
rs2900503
rs17032291
rs2998141
rs13223944
rs4717843
rs2074881
rs7712231
rs4270579

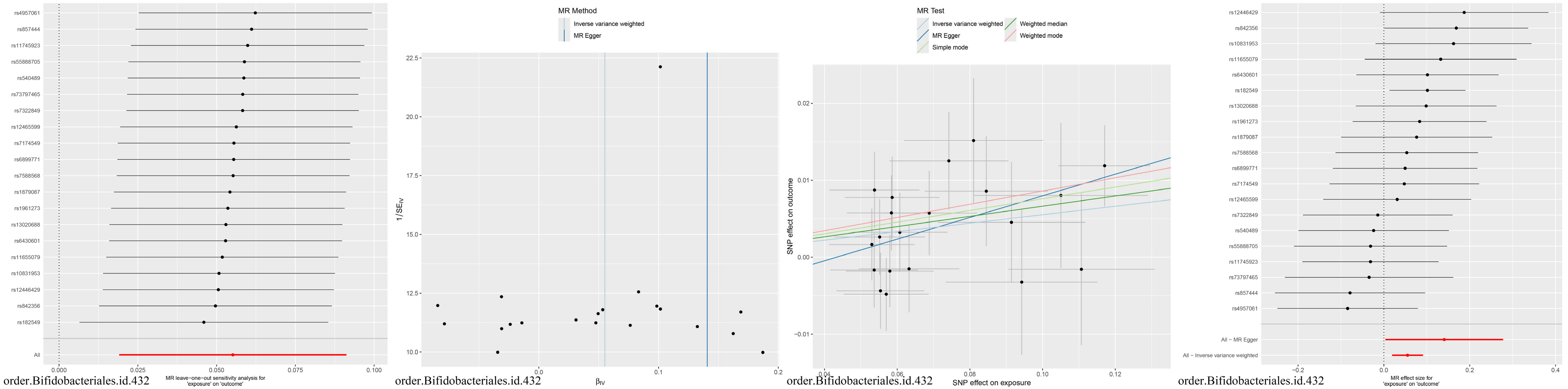
rs4957061
rs857444
rs11745923
rs55888705
rs540489
rs73797465
rs7322849
rs12465599
rs7174549
rs6899771
rs7588568
rs1879087
rs1961273
rs13020688
rs6430601
rs11655079
rs10831953
rs12446429
rs842356
rs182549
All
order.Bifidobacteriales.id.432
MR leave−one−out sensitivity analysis for
'exposure' on 'outcome'
MR Method
Inverse variance weighted
MR Egger
1/SE_IV
order.Bifidobacteriales.id.432
β_IV
MR Test
Inverse variance weighted
MR Egger
Simple mode
Weighted median
Weighted mode
SNP effect on outcome
order.Bifidobacteriales.id.432
SNP effect on exposure
rs12446429
rs842356
rs10831953
rs11655079
rs6430601
rs182549
rs13020688
rs1961273
rs1879087
rs7588568
rs6899771
rs7174549
rs12465599
rs7322849
rs540489
rs55888705
rs11745923
rs73797465
rs857444
rs4957061
All − MR Egger
All − Inverse variance weighted
order.Bifidobacteriales.id.432
MR effect size for
'exposure' on 'outcome'

F.

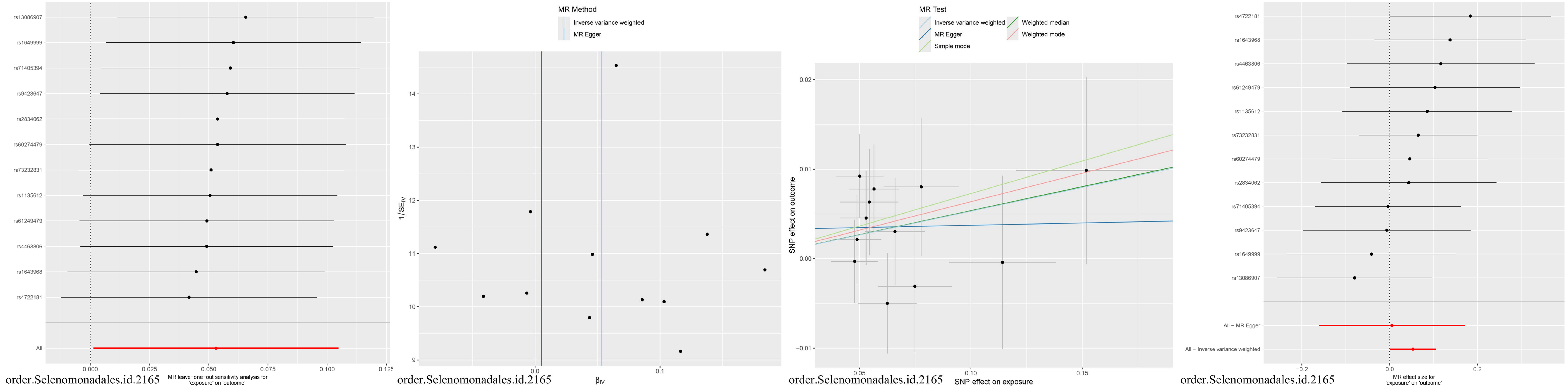
rs13086907
rs1649999
rs71405394
rs9423647
rs2834062
rs60274479
rs73232831
rs1135612
rs61249479
rs4463806
rs1643968
rs4722181
All
order.Selenomonadales.id.2165
MR leave−one−out sensitivity analysis for
'exposure' on 'outcome'
MR Method
Inverse variance weighted
MR Egger
1/SE_IV
order.Selenomonadales.id.2165
β_IV
MR Test
Inverse variance weighted
MR Egger
Simple mode
Weighted median
Weighted mode
SNP effect on outcome
order.Selenomonadales.id.2165
SNP effect on exposure
rs4722181
rs1643968
rs4463806
rs61249479
rs1135612
rs73232831
rs60274479
rs2834062
rs71405394
rs9423647
rs1649999
rs13086907
All − MR Egger
All − Inverse variance weighted
order.Selenomonadales.id.2165
MR effect size for
'exposure' on 'outcome'

G.

S2. Resultant plots of 5 suggestive inflammatory proteins associated with COVID-19. (A), Eukaryotic translation initiation factor 4E-binding protein 1 (B), Fibroblast growth factor 21 (C), Fibroblast growth factor 23 (D), Monocyte chemoattractant protein-1 (E), Thymic stromal lymphopoietin

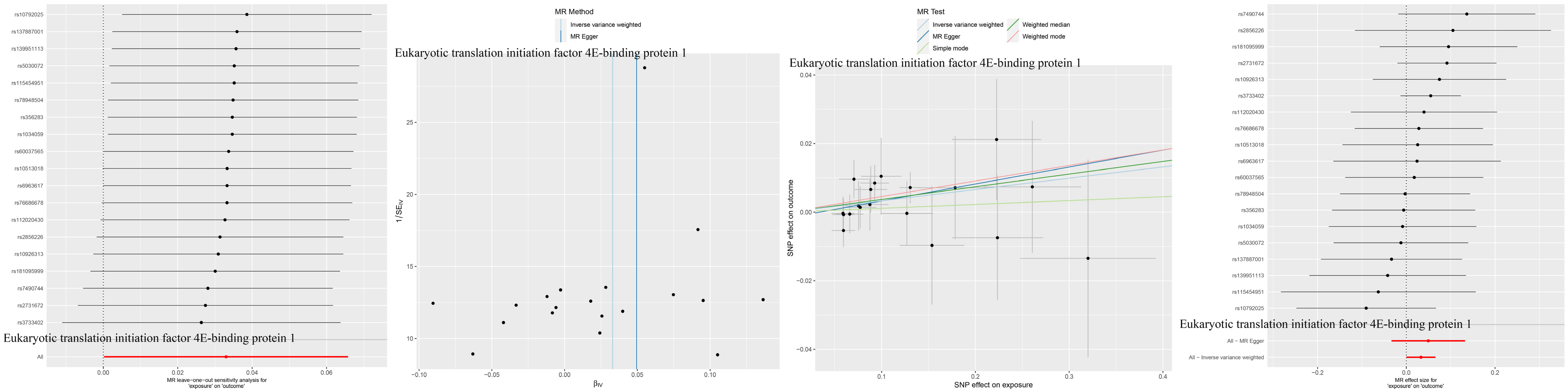

A.

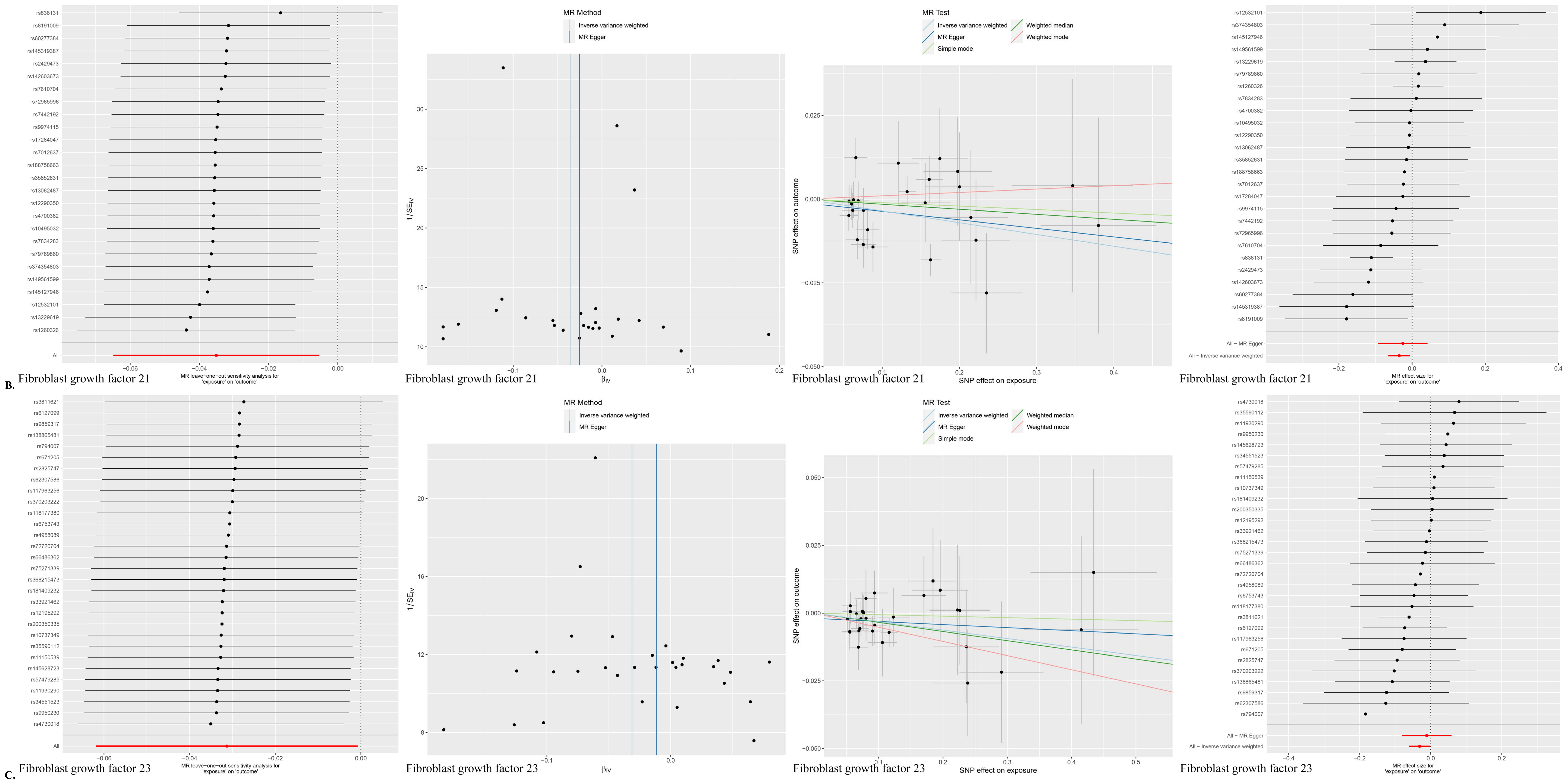
B.
Fibroblast growth factor 21
MR leave–one–out sensitivity analysis for 'exposure' on 'outcome'
All
Fibroblast growth factor 21
MR Method
Inverse variance weighted
MR Egger
1/SE$_{IV}$
β$_{IV}$
Fibroblast growth factor 21
MR Test
Inverse variance weighted
MR Egger
Simple mode
Weighted median
Weighted mode
SNP effect on outcome
SNP effect on exposure
Fibroblast growth factor 21
All – MR Egger
All – Inverse variance weighted
MR effect size for 'exposure' on 'outcome'
C.
Fibroblast growth factor 23
MR leave–one–out sensitivity analysis for 'exposure' on 'outcome'
All
Fibroblast growth factor 23
MR Method
Inverse variance weighted
MR Egger
1/SE$_{IV}$
β$_{IV}$
Fibroblast growth factor 23
MR Test
Inverse variance weighted
MR Egger
Simple mode
Weighted median
Weighted mode
SNP effect on outcome
SNP effect on exposure
Fibroblast growth factor 23
All – MR Egger
All – Inverse variance weighted
MR effect size for 'exposure' on 'outcome'

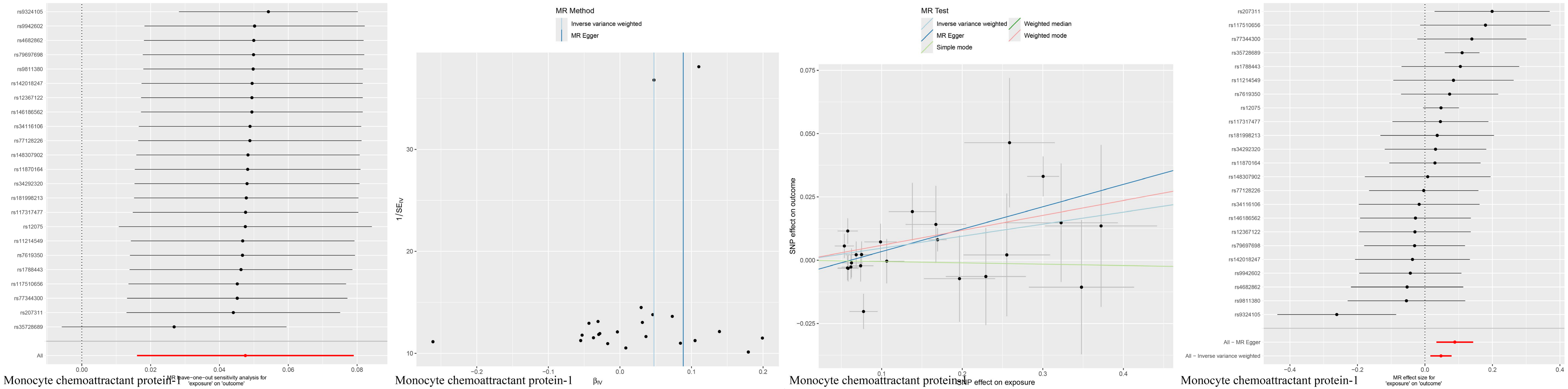
MR leave−one−out sensitivity analysis for 'exposure' on 'outcome'
All
MR Method
Inverse variance weighted
MR Egger
1/SE$_{IV}$
β$_{IV}$
MR Test
Inverse variance weighted
MR Egger
Simple mode
Weighted median
Weighted mode
SNP effect on outcome
SNP effect on exposure
All − MR Egger
All − Inverse variance weighted
MR effect size for 'exposure' on 'outcome'
Monocyte chemoattractant protein-1
Monocyte chemoattractant protein-1
Monocyte chemoattractant protein-1
Monocyte chemoattractant protein-1

D.

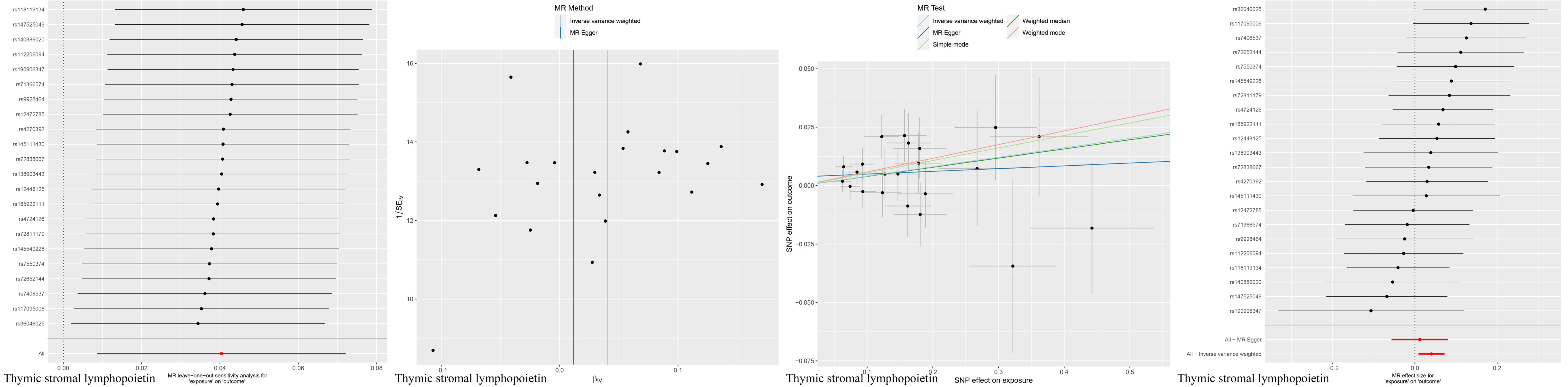
MR leave−one−out sensitivity analysis for 'exposure' on 'outcome'
All
MR Method
Inverse variance weighted
MR Egger
1/SE$_{IV}$
β$_{IV}$
MR Test
Inverse variance weighted
MR Egger
Simple mode
Weighted median
Weighted mode
SNP effect on outcome
SNP effect on exposure
All − MR Egger
All − Inverse variance weighted
MR effect size for 'exposure' on 'outcome'
Thymic stromal lymphopoietin
Thymic stromal lymphopoietin
Thymic stromal lymphopoietin
Thymic stromal lymphopoietin

E.
